# Microvesicle-Mediated Communication Within the Alveolar Space: Mechanisms of Uptake by Epithelial Cells and Alveolar Macrophages

**DOI:** 10.3389/fimmu.2022.853769

**Published:** 2022-04-27

**Authors:** Sanooj Soni, Kieran P. O’Dea, Eiko Abe, Maryam Khamdan, Sneh V. Shah, Padmini Sarathchandra, Michael R. Wilson, Masao Takata

**Affiliations:** ^1^ Division of Anaesthetics, Pain Medicine and Intensive Care, Faculty of Medicine, Imperial College London, Chelsea and Westminster Hospital, London, United Kingdom; ^2^ National Heart & Lung Institute, Imperial College London, Heart Science Centre, Harefield Hospital, Harefield, United Kingdom

**Keywords:** extracellular vesicles, microvesicle internalization, alveolar space, intercellular communication, microvesicle processing

## Abstract

Intra-alveolar microvesicles (MVs) are important mediators of inter-cellular communication within the alveolar space, and are key components in the pathophysiology of lung inflammation such as acute respiratory distress syndrome (ARDS). Despite the abundance of data detailing the pro-inflammatory effects of MVs, it remains unclear how MVs interact or signal with target cells in the alveolus. Using both *in vivo* and *in vitro* alveolar models, we analyzed the dynamics of MV uptake by resident alveolar cells: alveolar macrophages and epithelial cells. Under resting conditions, the overwhelming majority of MVs were taken up by alveolar macrophages. However, following lipopolysaccharide (LPS)-mediated inflammation, epithelial cells internalized significantly more MVs (p<0.01) whilst alveolar macrophage internalization was significantly reduced (p<0.01). We found that alveolar macrophages adopted a pro-inflammatory phenotype after internalizing MVs under resting conditions, but reduction of MV uptake following LPS pre-treatment was associated with loss of inflammatory phenotype. Instead, MVs induced significant epithelial cell inflammation following LPS pre-treatment, when MV internalization was most significant. Using pharmacological inhibitors, we interrogated the mechanisms of MV internalization to identify which endocytic pathways and cell surface receptors are involved. We demonstrated that epithelial cells are exclusively dependent on the clathrin and caveolin dependent endocytotic pathway, whereas alveolar macrophage uptake may involve a significant phagocytic component. Furthermore, alveolar macrophages predominantly engulf MVs *via* scavenger receptors whilst, epithelial cells internalize MVs *via* a phosphatidylserine/integrin receptor mediated pathway (specifically alpha V beta III), which can be inhibited with phosphatidylserine-binding protein (i.e. annexin V). In summary, we have undertaken a comprehensive evaluation of MV internalization within the alveolar space. Our results demonstrate that different environmental conditions can modulate MV internalization, with inflammatory stimuli strongly enhancing epithelial cell uptake of MVs and inducing epithelial cell activation. Our data reveal the unique mechanisms by which alveolar macrophages and epithelial cells internalize MVs thereby elucidating how MVs exert their pathophysiological effect during lung inflammation and injury. As MVs are potential novel therapeutic targets in conditions such as ARDS, these data provide crucial insights into the dynamics of MV-target cell interactions and highlight potential avenues for researchers to modulate and inhibit their pro-inflammatory actions within the alveolar space.

## Introduction

Microvesicles (MVs) are cell membrane-circumscribed extracellular particles, carrying a variety of molecular cargo, such as proteins, receptors and nucleic acids (1–3) over a distance to remote cells (1–3). They provide an alternative yet essential pathway for inter-cellular communication (2, 4) and have been implicated in the pathophysiology of various inflammatory diseases (5–8). This has led to a considerable amount of interest regarding the role of MVs in inflammatory lung diseases such as acute respiratory distress syndrome (ARDS). Indeed, we have previously demonstrated that intra-alveolar MVs, particularly alveolar macrophage-derived MVs, are potent initiators of acute lung injury (ALI), mediated by molecular cargo packaged within them (9, 10) and it is now evident that MVs are key components in the pathophysiology of lung inflammation as well as potential novel therapeutic targets (9, 11–13).

**Figure 1:**
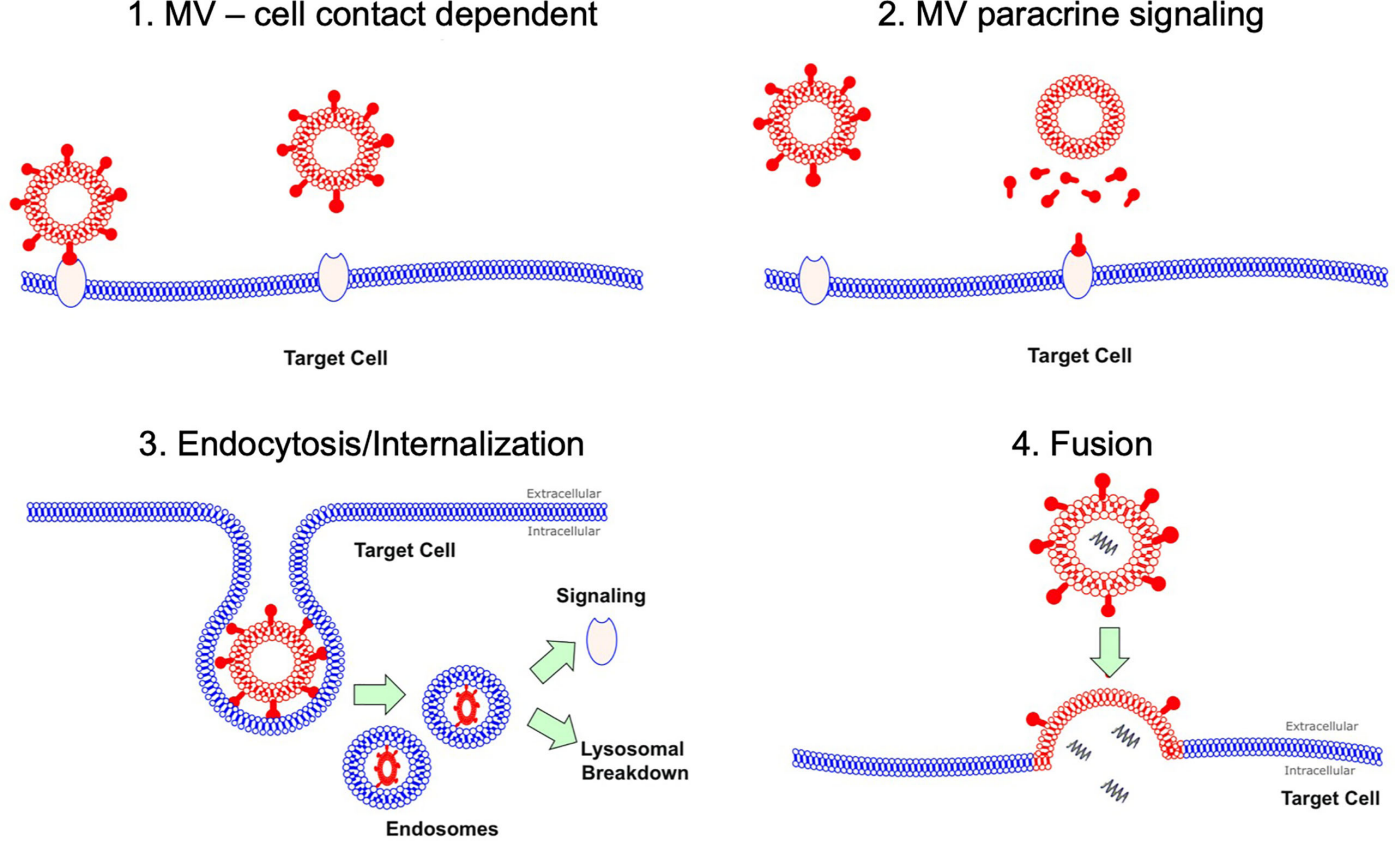
MV interaction with target cells. There are several hypotheses how MVs may interact with their target cells. Firstly this could be as simple MV and cell contact *via* a receptor *via* ligand-receptor interaction. Secondly this could occur through a paracrine fashion with MVs releasing soluble factors at a target cell site. MVs could undergo endocytosis, where they are internalized and then subsequently either undergo lysosomal degradation or activate endosomal signaling. Finally MV could fuse with their target cells resulting in fusion of 2 initially distinct membranes and subsequent release of contents into target cell cytoplasm.

Despite the abundance of data detailing the pro-inflammatory effects of MVs, it remains unclear how MVs interact or signal with target cells in the alveolus *in vivo*. Several mechanisms have been postulated based largely on extra-pulmonary models of inflammation (**Figure 1**): 1) *via* direct MV-cellular interaction forming a ligand-receptor complex (14); 2) through a paracrine fashion where MVs release their cargo near target cells, which subsequently act upon membrane receptors (15); 3) by endocytosis or internalization of MVs (and their cargo) by target cells (16, 17); and 4) through fusion of MVs with target cell membranes, thereby transferring their intra-vesicular cargo (18). Whist some of these data may be applicable to the alveolar space, it is important to note that within the unique environment of the alveolus, MVs may have prolonged or enhanced effects as the alveolus acts a semi-closed environment, protected from the rapid dilution or washout of MVs by blood flow. Therefore, the dynamics of MV communication in the alveolus are likely to differ from more commonly studied compartments such as the circulation.

To date, studies on MV interactions with alveolar cells have focused on the process of endocytosis. Alveolar macrophages, as resident professional phagocytes, internalize MVs resulting in lung inflammation (11), while a recent study has shown that insulin-like growth factor-1 (IGF-1) augments particle engulfment function of non-professional phagocytic cells such as epithelial cells, resulting in enhancement of MV uptake by airway epithelial cells *in vitro* (19). However these studies have focused on individual alveolar cells, predominantly in the *in vitro* setting and have not considered the alveolus as a whole, where alveolar macrophages and epithelial cells lie in close proximity to each other. This could lead to competition for uptake, which would not be apparent using isolated cells *in vitro*. Furthermore, it remains unknown whether differential uptake mechanisms/pathways occur between these different alveolar cells or if specific cellular populations preferentially take up MVs. Since MVs are a promising therapeutic avenue in lung inflammatory diseases such as ARDS, it is crucial to address these points but also to understand how MVs interact with these different cells in the alveolar environment.

In this study, we performed a comprehensive analysis of MV communication with target intra-alveolar cells in both *in vivo* and *in vitro* models, identifying key receptors and pathways involved in MV internalization in resting as well as inflammatory conditions. We showed that MV uptake and internalization in the alveolar space is performed primarily by alveolar macrophages rather than epithelial cells. However, following exposure to lipopolysaccharide (LPS), epithelial cell uptake is significantly increased, enhancing epithelial cell inflammation, whilst alveolar macrophage internalization of MVs is reduced. Furthermore, we demonstrated clear differences in MV uptake mechanism between these two cell types, i.e. MV uptake by alveolar macrophages is predominantly a scavenger receptor mediated process, whilst epithelial cells rely upon integrin receptors. These data provide crucial mechanistic information and delineate potential means to interrupt MV-mediated signaling in the alveolus for therapeutic purposes.

## Materials and Methods

### Animal Experiments

All protocols were approved by the Ethical Review Board of Imperial College London, carried out under the authority of the UK Home Office in accordance with the Animals (Scientific Procedures) Act 1986, UK and reported in compliance with the ARRIVE guidelines. One hundred and fifty-six male C57BL/6 mice (Charles River, Margate, UK), aged 10-14 weeks were used. Mice were housed in individual ventilated cages (maximum number of 5 per cage) and exposed to 12-hour light and dark cycles. All experiments were initiated and completed during the light cycle and no unexpected adverse effects were observed in any of the treatment groups.

### *In Vitro* MV Production and Fluorescent Labeling

RAW 264.7 macrophages cells (Sigma-Aldrich, UK) were washed and pre-treated with 1μg/ml of ‘Ultrapure’ lipopolysaccharide (LPS) (*In vivo*gen Toulouse, strain: E. coli O111:B4) for 1 hour to induce inflammatory conditions as previously described (9, 10). Cells were then stimulated with 3mM of ATP disodium salt (Bio-techne, UK) to induce release of ‘pro-inflammatory’ MVs. Supernatants were collected, centrifuged to remove cells (200g 10 minutes at 4°C) and then labeled with 5μM of 1,1’-Dioctadecyl-3,3,3’,3’-Tetramethylindodicarbocyanine Perchlorate (DiD) (ThermoFisher Scientific, UK) in Diluent C, in dark at room temperature for 7 minutes. DiD-labeled MVs were then pelleted (20,000*g* for 30 min at 4°C) and washed twice to remove unbound dye. The relative fluorescence of MVs was assessed using a fluorescence plate reader (Bio-tek FLX 800; Bio-tek instruments, USA), and a standardized amount of fluorescent MVs (25,000 relative fluorescence units (RFU), which corresponds to approximately 1 x 10^6^ MVs) was then added to our *in vitro* or *in vivo* models [**Supplementary Figures 1**, **2** (20)]. This dose of MVs was chosen as we have previously observed up to approximately 1 x 10^6^/ml of alveolar macrophage-derived MVs in bronchoalveolar lavage fluid (BALF) samples in i.t. LPS-induced ALI in mice at 1 hour (9).

**Figure 2:**
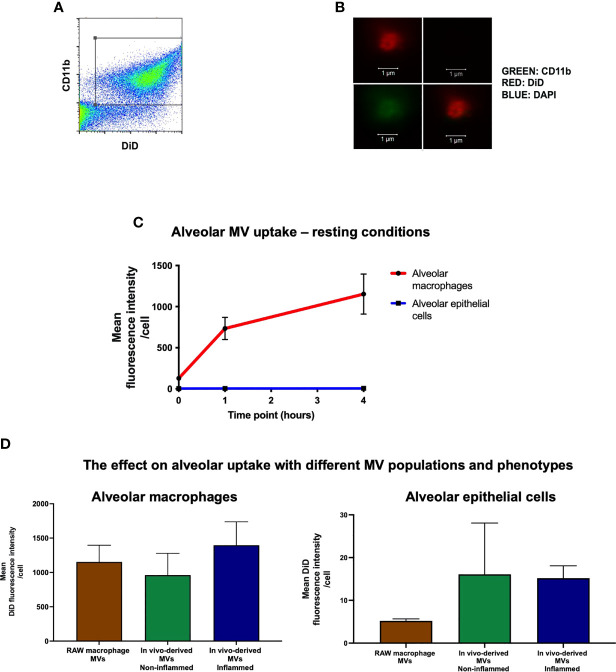
*In vivo* uptake of MVs. **(A)** Flow cytometry plot of RAW macrophage derived MVs, demonstrating that DiD labeled events were also positive for CD11b, confirming their identity as macrophage-derived MVs. **(B)** These particles were readily visualized by confocal microscopy as DiD (red, top left panel), particles negative for nuclear materials (DAPI^-^, top-right), but positive for CD11b (green, bottom-left, co-localization shown in the bottom-right combined image) (n = 3). **(C)** DiD labeled RAW macrophage MVs (25,000 RFU) were instilled into the trachea of untreated mice and *in vivo* MV uptake by alveolar macrophages and epithelial cells was assessed by flow cytometry at 1 and 4 hours. Time 0 represents baseline auto-fluorescence, i.e. no MVs added. Alveolar macrophages significantly internalized the majority of MVs compared to alveolar epithelial cells at both time points [1 hour: alveolar macrophage 734 ± 135 RFU vs. epithelial cells 4.78 ± 0.57 RFU; 4 hours: alveolar macrophage 1153 ± 243 RFU vs. epithelial cells 5.21 ± 0.47 RFU (n = 5)]. **(D)** Alveolar macrophages, as compared to epithelial cells, internalized the majority of MVs at 1 hour after installation *in vivo* regardless of the MV phenotypes used: 1) *in vitro*-derived RAW macrophage MVs (i.e. from stimulated RAW macrophage culture); 2) *in vivo*-derived MVs, non-inflamed (intra-alveolar MVs harvested by lung lavage from untreated mice); and 3) *in vivo*-derived MVs, inflamed (intra-alveolar MVs harvested by lung lavage from LPS-treated mice). (n = 3-6, not significant by one way ANOVA).

MVs were identified by flow cytometry (CyAn™ ADP flow cytometer, Beckman Coulter, UK) as events under 1μm in size (forward scatter and side scatter with a trigger threshold of 0.01 were used to elucidate a 1µm gate that was delineated using sizing beads) and positive for specific surface marker CD11b (M1/70; Biolegend, CA) and DiD (**Figure 2A**). MVs were also enumerated using Accucheck counting beads (Invitrogen, Paisley, UK) as previously shown (9, 10, 20). Data were analysed using FlowJo software. All stained MV samples were also treated with 0.1% triton detergent in order to correctly differentiate MVs from non-vesicular antibody-bound events (21). The centrifugation and flow cytometry methods to isolate and characterise MVs in this study have been previously validated both by high-resolution imaging and electron microscopy (10).

### *In Vivo* MV Production

*In vivo*-derived MVs were harvested from our LPS model of ALI as previously described (composed primarily of alveolar macrophage and epithelial cell-derived MVs) (9). In brief, mice were anesthetized (intraperitoneal ketamine 90mg/kg; xylazine 10mg/kg) and 20µg LPS in 50µl was instilled intratracheally (i.t.). After 1 hour, animals were euthanized and tracheostomized, BALF were obtained by flushing and gently aspirating 700μl of 0.9% saline in and out of the lungs *via* the endotracheal tube three times, and centrifuged to remove cells and larger particles (200g, 10mins at 4°C). Cell free supernatants were then stained with DiD as described above. DiD-labeled *in vivo* generated MVs were then pelleted (20,000g for 30 min at 4°C) and washed twice to remove unbound dye and other stimulatory factors.

### *In Vivo* Model of Intra-Alveolar MV Uptake

Fluorescent-labeled RAW macrophage-derived MVs (25,000 RFU, resuspended in 50µl normal saline) or post-wash supernatant (50µl of supernatant following second wash step as control) was instilled i.t. into the lungs of randomly selected mice by an investigator blinded to the treatment groups (**Supplementary Figure 1**). In a separate set of experiments, *in vivo* derived MVs (25,000 RFU, obtained as described above) were also instilled i.t. into the lungs of randomly selected mice (**Supplementary Figure 2**). Either 1 or 4 hours after instillation, mice were euthanized, lungs were removed and mechanically disrupted in warm fixation buffer using a GentleMACS dissociator [Miltenyi Biotec, Surrey, UK (20)]. Samples were then passed through 40µm sieves, washed and resuspended twice in flow cytometry buffer (2% fetal calf serum, 2mM EDTA and 0.1% sodium azide constituted in PBS) to yield a fixed single cell suspension. Uptake was then evaluated by assessing mean fluorescence intensity (MFI) of DiD in alveolar cells by flow cytometry. As described previously (9), alveolar macrophages were identified as CD45^+^ (clone 30-F11; Biolegend), CD11c^+^ (clone N418; eBioscience, CA), CD11b^-^, F4/80^+^ (BM8; eBioscience). Epithelial cells were identified as CD45^-^, CD31^-^ (MEC 13.3; BD Bioscience, CA), and T1alpha^+^ (8.1.1; Biolegend) events (9). In some experiments, mice were pre-treated with i.t. 20ng LPS (in 50µl normal saline) or saline (50 µl) for 1 hour in order to assess the effect of underlying lung inflammation on MV uptake.

### *In Vitro* Model of Intra-Alveolar MV Uptake

In order to model the intra-alveolar environment *in vitro*, we created a co-culture system comprised of primary alveolar macrophages and Murine Lung Epithelial (MLE-12) cells (ATCC, UK). MLE-12 cells were seeded overnight in a 24 well-plate at a density of 10^5^ cells/well and primary alveolar macrophages [harvested by lung lavage from untreated mice as described previously (9)] were added to MLE cells for one hour in the ratio of 1:5 (22). DiD-labeled RAW macrophage-derived MVs or post-wash supernatant were then incubated in this *in vitro* model of the alveolus. After 1 or 4 hours, cells were detached using EDTA containing solution (Versene, Life Technologies), stained with the fluorescently conjugated antibodies against CD45, CD11c and T1α and DiD MFI of individual cells were assessed.

### Mechanisms of MV Uptake

In our *in vitro* alveolar co-culture model, we examined the surface expression of variety of scavenger/integrin receptors in each cell type *via* flow cytometry: MERTK (2B10C42; biolegend), TIM4 (RMT4-54; biolegend), Alpha V Beta III (2C9.G2; Biolegend), Alpha V Beta V (RMV-7; Biolegend), Macrophage Scavenger Receptor 1 (REA148; Miltenyi), MARCO, CD36 (HM36; Biolegend); CD68 (FA-11; Biolegend).

In separate experiments, to assess mechanism of MVs internalization, we pre-incubated the cells in the alveolar co-culture model with 2µM Cytochalasin D or 0.25mM Dynasore prior to treatment with DiD-labeled RAW MVs. DiD fluorescence was then assessed in these cells as described above.

### Confocal Microscopy

Primary alveolar macrophages and MLE cells were seeded on coverslips and then incubated with DiD-labeled RAW MVs for 1 hour. In separate experiments, to visualize DiD-labeled MVs *via* confocal microscopy, MVs were placed on poly-L-ornithine coated coverslips to encourage adherence for 6 hours. Thereafter both MVs and cells were washed, fixed, permeabilized with 0.5% triton-X 100 and incubated with 3% bovine serum albumin (Sigma-Aldrich) for 30mins. Slides were then incubated with 5µg/ml T1 alpha (ab109059; Abcam, Cambridge UK) or 5µg/ml CD45 (ab23910; Abcam) overnight in the dark at 4°C, followed by washing and incubation with secondary antibodies (1:1000) for 1 hour. After washing, slides were treated with 4’,6-diamidino-2-phenylindole (DAPI) intra-nuclear stain (1:10000) solution (pre-made) for 10mins. Coverslips were placed on slides with Mounting PermaFluor (ThermoFisher Scientific) and viewed using a Zeiss LSM880 NLO multiphoton confocal imaging system with Axio Observer 1 microscope. The objective lens used was a Plan Apochromat 40x/1.3 oil DIC UVVIS-IR. The imaging medium was oil and the temperature -20°C. The fluorochromes used were Alexa-Fluor 488 and Alexa-Fluor 594. CZI Images were acquired using Zen software, which were then exported as 16 bit Tiff images. No image processing software was used.

### Statistical Analysis

Shapiro-Wilk normality tests were carried out (IBM SPSS). Comparisons between two data sets were performed using either paired T-tests or Wilcoxon Rank Sum test. All data was analyzed on GraphPad Prism and are expressed as Mean ± SD, or Median ± Interquartile range. A p<0.05 was defined as the minimum threshold for statistical significance.

## Results

### *In Vivo* Uptake of MVs

We have previously shown that ‘pro-inflammatory’ macrophage-derived MVs are rapidly released within the alveolus *in vivo* and involved in the early pathogenesis of ALI (9). To model this effect, RAW macrophages were stimulated with LPS (inflammatory stimulus) followed by ATP (danger signal) to induce release of pro-inflammatory MVs, which were then labeled with the fluorescence lipophilic dye DiD (17) for detection by flow cytometry and confocal microscopy (**Figures 2A, B**). A known fluorescent quantity of these MVs [25,000 RFU which corresponds to 1 x 10^6^ MVs; we previously found up to approximately 1 x 10^6^/ml of alveolar macrophage-derived MVs in BALF in i.t. LPS-induced ALI in mice at 1 hour (9)] was instilled into the trachea of untreated mice, and MV uptake by different alveolar cells was assessed by flow cytometric analysis of lung single cell suspensions. One hour after instillation, we found that alveolar macrophages rather than epithelial cells internalized the majority of MVs (**Figure 2C**), despite several studies previously demonstrating that epithelial cells rapidly take up MVs (19, 23, 24). This difference persisted over time, such that at 4 hours alveolar macrophage still internalized the majority of MVs (1153 ± 244 RFU for alveolar macrophages vs. 5.21 ± 0.47 RFU for epithelial cells).

To assess the uptake of the mixed populations of MVs released *in vivo* within the alveolus, we used intra-alveolar MVs harvested from an *in vivo* model of ALI. These MVs contained ‘mixed’ populations, predominantly composed of alveolar macrophage- and epithelial cell-derived MVs (with concentrations of ~1000 MVs/µL and ~650 MVs/µL respectively in the original BALF samples), and have significant pro-inflammatory activity (9). We also used intra-alveolar MVs harvested from untreated mice as a control, which are composed of both alveolar macrophage- and epithelial cell-derived MVs (~300 and ~180 MVs/µL in the original BALF samples), and devoid of inflammatory activity. Both types of MVs were labelled and instilled i.t. into another mice (**Supplementary Figure 2**). Alveolar macrophages still internalized the overwhelming majority of these primary, *in vivo*-generated ‘inflamed’ or ‘non-inflamed’ MVs compared to epithelial cells 1 hour after installation (**Figure 2D**). Interestingly, there was a trend that epithelial cells internalized more *in vivo* derived MVs compared to RAW MVs but this did not reach statistical significance (epithelial uptake of RAW MV: 5.21 ± 0.47 RFU vs. non-inflamed *in vivo* MVs: 16.1 ± 12.0 RFU vs. inflamed *in vivo* MVs: 15.9 ± 2.9 RFU). Nevertheless, these data suggest that irrespective of MV population (either alveolar macrophage- or epithelial cell-derived MVs) and phenotype (inflamed or no-inflamed), alveolar macrophages internalize the majority of MVs within the alveolar space under normal physiological conditions, within the time frame of our observations.

### *In Vitro* Uptake of MVs

**Figure 3:**
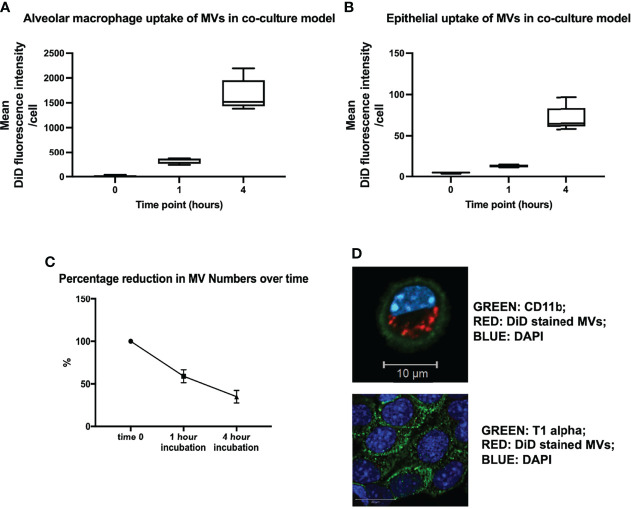
**(A)** *In vitro* uptake of DiD labeled uptake in our in vitro alveolar system. Under resting conditions, a known quantity of raw cell derived-MVs (25,000 RFU) were introduced into our in vitro alveolar system consisting of alveolar macrophages and MLE cells at a ratio of 1:5. Substantial amount of MVs were internalized by alveolar macrophage rather than MLEs cells at either 1 or 4 hours. **(B)** Epithelial cells did take up MVs albeit to a much lesser extent compared to alveolar macrophages, with a rise in uptake after 4 hours. **(C)** The amount of MVs, recovered from our co-culture system decreased over time, in keeping with increased uptake of MVs by the cells in the co-culture system. **(D)** Confocal microscopy of MV uptake by alveolar cells after 1 hour incubation. The green dye represents cell surface integrin CD11b, which depicts the cell membrane. Our image demonstrates the presence of DiD stained MVs clearly within the membrane (likely to be localized to the cytoplasm) of the macrophage confirming our flow cytometry results. However MLE cells (bottom) take up a very small amount of MVs, as minimal amounts of red stained MVs are present within these cells (n = 3). Parametric or non-parametric data displayed as mean ± s.d. or box–whisker plots showing the median, IQR and minimum/maximum values respectively (**A–C** experiments n = 5-6).

In order to model the environment with the lungs, we created an *in vitro* alveolar system composed of primary alveolar macrophages (obtained from untreated mice) and murine lung epithelial (MLE-12) cells (22). This co-culture system allowed us to confirm our *in vivo* results and explore the dynamics and mechanisms of MV-mediated communication with alveolar cells in detail. DiD labeled RAW-derived MVs were incubated within this *in vitro* environment for 1 or 4 hours, and the uptake of MVs by individual cells was measured by flow cytometry and confocal microscopy. As occurred *in vivo*, substantial amounts of MVs were internalized by alveolar macrophages, after both 1 and 4 hours incubation time (**Figure 3A**), while epithelial cells did internalize MVs but this process occurred to a much lesser extent compared to alveolar macrophages (**Figure 3B**). As would be expected from this *in vitro* alveolar model (which is essentially a completely closed system), the amount of MVs recovered in the cell culture supernatant substantively decreased over time, consistent with these MVs being taken up by alveolar cells (**Figure 3C**). These findings were also confirmed by confocal microscopy (**Figure 3D**), where alveolar macrophages can be seen to internalize MVs.

Interestingly, we found that when we increased MV numbers in our co-culture system, alveolar macrophages appeared to become saturated and unable to internalize any further MVs. Instead, epithelial cells started taking up MVs to almost a similar extent as alveolar macrophages at 4 hours of incubation, presumably as there is less competition from alveolar macrophages (**Supplementary Figure 3**). There may also be increased uptake efficiency of the epithelial cells at higher MV concentrations. However, it is important to note that the number of MVs required to precipitate this effect (0.5 to 1.5 x 10^7^/ml) far exceeds the number of macrophage MVs measured in BALF (~1 x 10^6^/ml) in our *in vivo* models of ALI (9).

### Effects of Inflammation on MV Internalization

**Figure 4:**
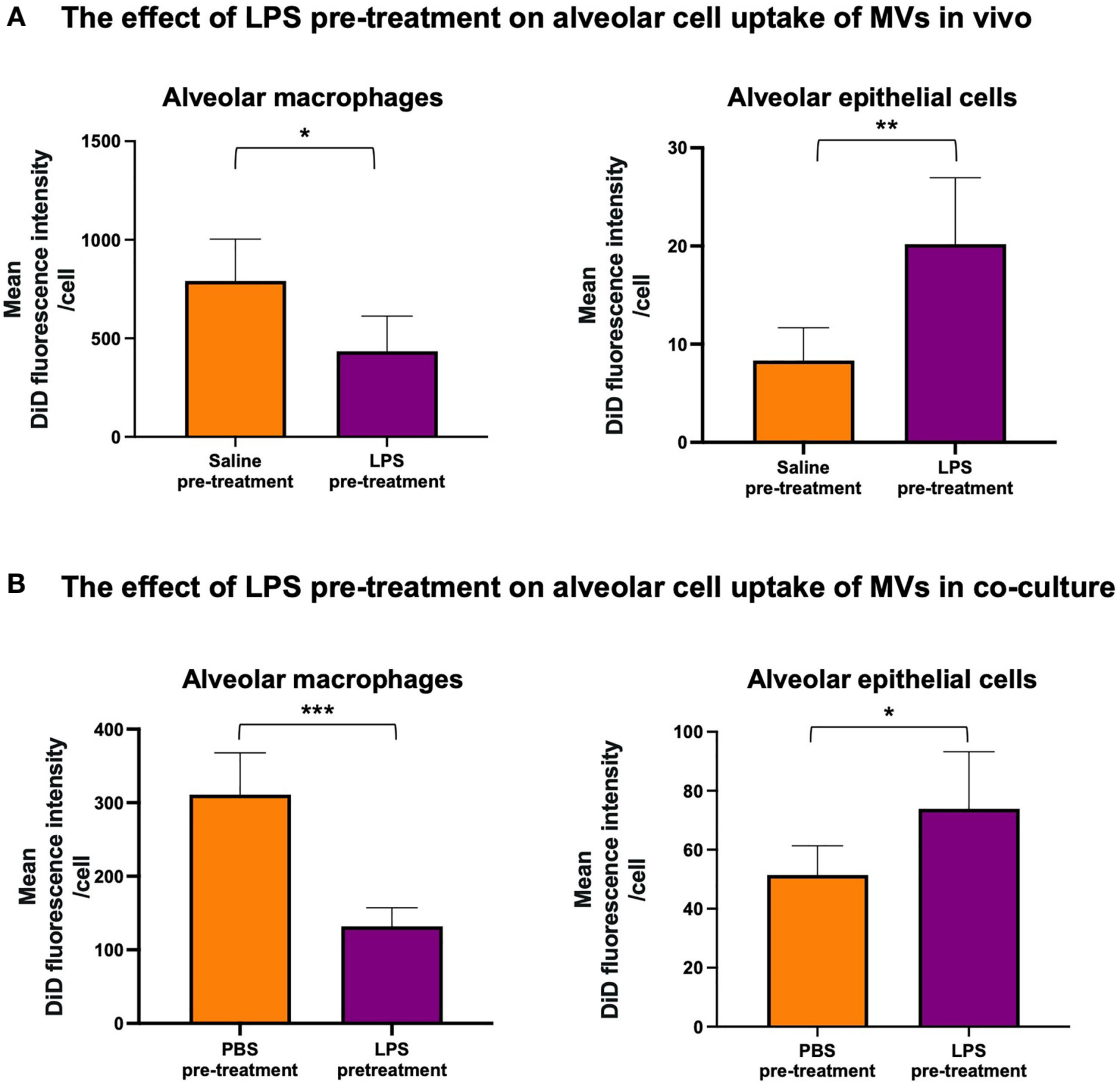
The effect of LPS on internalization of RAW macrophage derived MVs by alveolar cells. **(A)** 20ng LPS pre-treatment (via intra-tracheal instillation) caused a decrease in MV uptake by alveolar macrophages (left), but produced a marked increase in MV uptake by epithelial cells (right). **(B)** This phenomenon was repeated *in vitro* such that pre-treatment with 1µg/ml LPS for 1 hour caused a significant decrease in MV uptake by alveolar macrophages (left) but a significant increase in epithelial cell uptake (right). Parametric data displayed as mean ± s.d. *p < 0.05, **p < 0.01, ***p < 0.001 (all experiments n=5).

Next, we assessed whether MV internalization in the alveolar space would differ during inflammation. DiD-labeled RAW macrophage-derived MVs were instilled into the trachea of mice that were pre-treated with either 20ng LPS or saline. We found that LPS pre-treatment caused a significant switch in MV uptake: alveolar macrophage internalization was reduced (~<50%, p<0.01) whilst epithelial cell uptake was increased (~>150%, p<0.01) (**Figure 4A**). This pattern was replicated in our *in vitro* model when the co-culture was pretreated with 1µg/ml LPS (**Figure 4B**). These findings demonstrated that inflammatory conditions substantively modulate MV uptake within the alveolar space, producing a partial shift of MV uptake/internalization from alveolar macrophages to epithelial cells.

**Figure 5:**
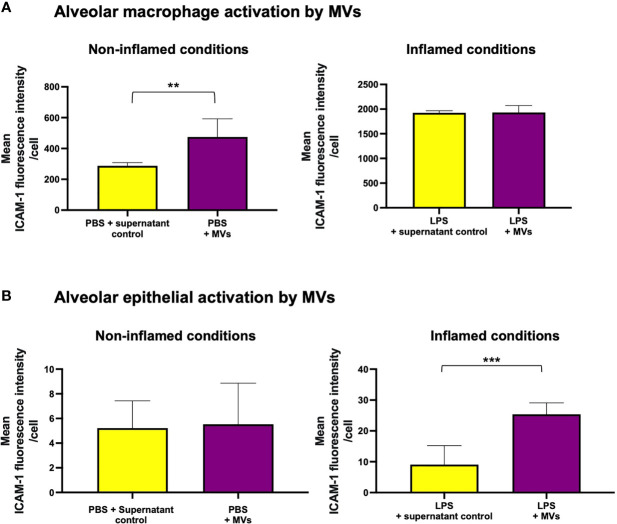
MV mediated inflammation within our *in vitro* alveolar system. (**A**, left) Under resting conditions, addition of RAW macrophage derived MVs caused a significant increase in ICAM-1 expression on alveolar macrophages. (**A**, right) However, when exposed to 1µg/ml LPS (where MV uptake is substantively reduced), MVs no longer have a pro-inflammatory effect on alveolar macrophages. **(B)** However this trend is reversed within epithelial cells when ICAM-1 expression significantly increases only in epithelial cells that have been pre-treated with LPS (where MV uptake is maximal). Parametric data displayed as mean ± s.d. **p < 0.01, ***p < 0.001. (all experiments n = 5).

We also assessed whether MV uptake caused a change in alveolar cell phenotype or activation status. Using ICAM-1 as a surrogate marker of cell activation (25, 26), we found that alveolar macrophages adopted a pro-inflammatory phenotype after internalizing RAW macrophage-derived MVs under resting conditions (p<0.01, **Figure 5A**). Interestingly, when uptake was reduced following pre-treatment with 1µg/ml LPS, the pro-inflammatory effect of MVs was no longer evident (**Figure 5A**). Conversely, MVs only induced significant epithelial cell activation following pre-treatment with LPS, when MV internalization was most significant (**Figure 5B**).

### Endocytosis of MVs

**Figure 6:**
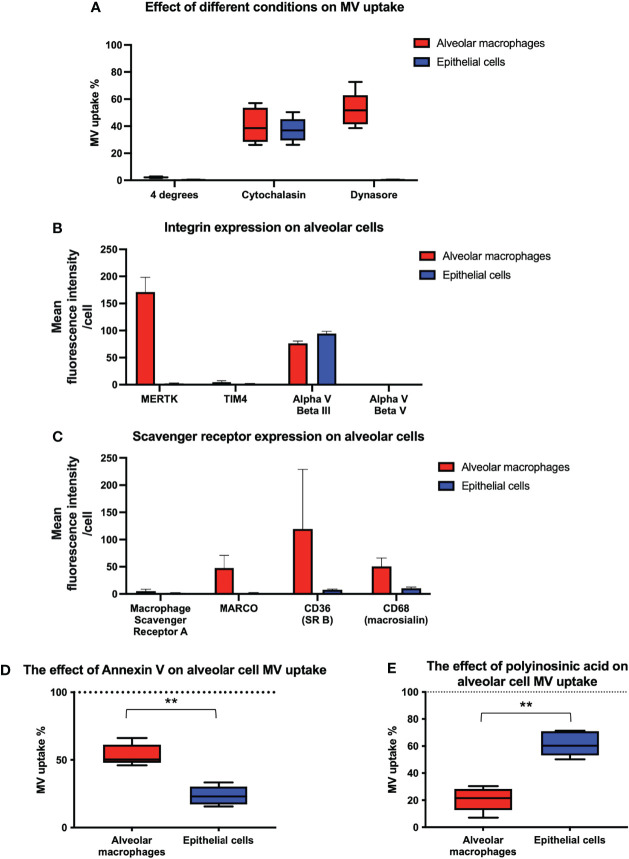
Inhibition of endocytosis in alveolar cells. **(A)** Performing experiments at 4°C completely obliterated MV engulfment in both alveolar macrophages and epithelial cells. When our co-culture system was pre-incubated with either cytochalasin or dynasore, endocytosis was significantly inhibited in both cell types. However dynasore had a greater effect upon epithelial cells, completely abrogating internalization, which may have some mechanistic significance. Results expressed as a percentage of control MV uptake (compared to experiments where no inhibitor used). **(B)** Flow cytometry analysis of integrins on the surface of alveolar macrophages and MLE cells. Alveolar macrophages express MERTK and Alpha V Beta III integrin receptors on their surface whilst epithelial cells only express Alpha V Beta III. **(C)** Flow cytometry analysis of scavenger receptors on the surface of alveolar macrophages and MLE cells. Alveolar macrophages express a variety of scavenger receptors including MARCO, CD36 and CD68 whereas epithelial cells do not. **(D)** The effect of blocking integrin on alveolar cell uptake. MVs were pre-incubated with annexin V prior to alveolar cells treatment. Annexin V binding to PS on surface of MVs and subsequent integrin receptor blockade, had a significant effect on epithelial cell blockade compared to alveolar macrophages, although alveolar macrophage uptake was also inhibited. Results expressed as a percentage of control MV uptake (where no annexin V used). **(E)** The effect of blocking scavenger receptors on alveolar cell uptake. Our alveolar cells *in vitro* were pre-incubated with polyinosinic acid, a scavenger receptor inhibitor, prior to MV treatment. This significantly abrogated alveolar macrophage engulfment whilst have little effect upon epithelial cells. Results expressed as a percentage of control MV uptake (where polycytidylic acid used rather than polyinosinic acid). Results expressed as mean fluorescence intensity. Parametric data displayed as mean ± s.d. and non-parametric data displayed as box–whisker plots showing the median, IQR and minimum/maximum values. **p < 0.01 All experiments n = 5.

We then investigated the mechanisms of MV internalization in both alveolar macrophages and epithelial cells. Firstly, MV uptake experiments were carried out at 4°C which abolished MV uptake in both alveolar macrophages and epithelial cells, indicating that an active, energy dependent process is involved (**Figure 6A**). We next assessed the effect of inhibitors of endocytosis on MV uptake by each of these cells. Cytochalasin (generalized inhibitor of endocytosis) reduced alveolar macrophage uptake by 59.5%, and dynasore (cell-permeable inhibitor of the clathrin and caveolin dependent endocytosis pathway) inhibited macrophage uptake by 47.9% (**Figure 6A**). In contrast, epithelial cell uptake was inhibited by 62.7% by cytochalasin but completely blocked by dynasore (**Figure 6A**). The absolute effects of dynasore on epithelial cells, but not on alveolar macrophages, suggests that epithelial cells are exclusively dependent on the clathrin and caveolin dependent endocytotic pathway, whereas alveolar macrophage uptake may involve a significant phagocytic component.

### Receptor-Mediated Mechanism of MV Uptake

To identify receptor-mediated mechanisms involved in MV internalization by alveolar cells, we examined the presence of cell surface receptors that have been implicated in the uptake of endogenous and foreign particles or apoptotic cells, and thus may be involved in MV uptake. In particular, we investigated the relative expression of integrin receptors which bind to phosphatidylserine (PS) such as MerTK (27, 28), alpha V beta III (29, 30) and alpha V beta V (31), and scavenger receptors such as macrophage scavenger receptor A (32), MARCO (33), CD36 (34) and CD68 (35). We also studied the expression of the integrin TIM4 since it is expressed on the surface of macrophages and has a role in MV and exosome internalization (36, 37). We found that alveolar macrophages express MerTK and Alpha V beta III integrin receptors whereas epithelial cells only express Alpha V beta III integrin receptor (**Figure 6B**). In addition, we detected a variety of scavenger receptors on alveolar macrophages including MARCO, CD36 and CD68, but expression of scavenger receptors was low or undetectable on epithelial cells (**Figure 6C**). Based on these profiles, we hypothesized that both integrin (MerTK and alpha V beta III) and scavenger receptors (MARCO, CD36, and CD68) were involved in MV uptake by alveolar macrophages, whereas epithelial cell uptake occurred principally *via* integrin receptors (specifically alpha V beta III).

To examine this hypothesis, we pre-treated our co-culture system with either polyinosinic acid (50µg/mL) (Class A scavenger receptor inhibitor) or annexin V [binds to PS expressed on MVs preventing integrin/PS mediated MV uptake (38, 39)] and assessed their effect on MV uptake by each cells (**Figures 6D, E**). As predicted, annexin V inhibited uptake by both alveolar macrophages and epithelial cells but had a greater effect on uptake by epithelial cells. Conversely, scavenger receptor inhibitor polyinosinic acid dramatically reduced alveolar macrophage internalization of MVs, with a lesser effect on epithelial cell uptake. The results suggest that integrin receptors play an essential role in MV uptake by epithelial cells, whereas scavenger receptors play a more predominant role in that by alveolar macrophages.

## Discussion

Elevated production of MVs is a common feature in the pathophysiology of alveolar inflammation, particularly in conditions such as ARDS (9) or COPD (8), yet there is a paucity of data describing MV communication or cellular uptake within the alveolus. This study demonstrates several novel findings detailing MV trafficking within the unique environment of the alveolar space, under both resting and inflammatory states. We have provided convincing data that the overwhelming majority of MV uptake is performed by alveolar macrophages under resting conditions, irrespective of MV phenotype or origin and inducing a pro-inflammatory phenotype in these cells. Although epithelial cells internalized MVs only to a limited extent, this was substantively enhanced by LPS priming, leading to significant MV-induced epithelial cell activation. Furthermore, our data indicate that MVs are internalized by distinct receptor-mediated pathways: alveolar macrophages predominantly internalize MVs *via* scavenger receptors, whilst epithelial cells endocytose MVs through a PS/integrin receptor mediated pathway.

Within the alveolus, the major resident cell populations directly exposed to the air space are alveolar macrophages and epithelial cells. Previous studies investigating MV mediated communication within the alveolus have been performed mostly in an *in vitro* setting, concentrating on MV uptake by either epithelial cells (19, 23, 24) or alveolar macrophages (11, 28) alone. This is the first study to analyze MV internalization by different alveolar cells ‘*in vivo*’, and we have shown that the degree of uptake by each of these alveolar cells is dependent on the environmental condition to which they are exposed. Under normal physiological conditions, alveolar macrophages internalize the overwhelming majority of MVs, inducing an inflammatory phenotype, while epithelial cells internalize only a very modest proportion of MVs. Hence, in non-inflamed alveoli, any pro-inflammatory effects of MVs seems to be mediated largely by alveolar macrophages. However, in reality, MVs are actively produced within the alveolus when cells are exposed to injury/inflammation causing release of danger associated molecular patterns such as ATP (40, 41). Therefore, any study examining MV pro-inflammatory signaling in the alveolus should rather be conducted under inflammatory stress to better simulate clinically-relevant disease states. We found that during inflammation, alveolar macrophage uptake of MVs is substantively reduced, with the pro-inflammatory effect of MVs upon macrophages being no longer evident. In sharp contrast, MV uptake by alveolar epithelial cells was markedly enhanced, inducing significant epithelial cell activation. We have previously observed a similar phenomenon of uptake shift of circulating MVs such that LPS injection reduced MV uptake by liver Kupfer cells but enhanced internalization of MVs by monocytes marginated within the pulmonary vasculature, leading to significant pulmonary vascular inflammation (20). Previous studies have demonstrated the reduced expression of scavenger receptors such as CD36 and Marco on the surface of inflamed/M1-polarized macrophages, potentially explaining this finding (42, 43).

The mechanisms detailing MV communication and interaction with target cells remain poorly understood and this is particularly true within the alveolus during inflammatory lung diseases. It is thought that this interaction occurs primarily through endocytosis (3, 23), which consists of a number of pathways including macropinocytosis, phagocytosis, clathrin-dependent, caveolin-dependent, or clathrin/caveolin-independent pathways. Alveolar macrophages and epithelial cells are likely to internalize MVs *via* different endocytosis pathways (44, 45), and we undertook a series of experiments to identify the mechanisms and receptors involved in MV uptake by these alveolar cells. Our confocal microscopy images demonstrated that MVs are internalized by alveolar macrophages rather than fusion with cell membranes. This internalization is an active process, and cytochalasin D, an actin polymerization inhibitor and a generalized suppressor of endocytosis (38, 46), reduced but did not completely abolish MV uptake in both cell types to a similar extent as previously reported (47). Dynasore, a dynamin inhibitor which inhibits both clathrin and caveolin-dependent endocytosis (48), had a much more profound effect on epithelial cells, effectively abolishing epithelial cell uptake. This suggests that there are mechanistic differences in MV uptake between the two cell types: epithelial cell uptake is dependent upon clathrin and caveolin-dependent endocytosis whereas alveolar macrophages may be more reliant upon other endocytic mechanisms (e.g macropinocytosis and phagocytosis), which is consistent with recent data of endocytosis mechanisms of nanoparticle uptake by alveolar cells (49). Indeed, the different speed and capacity of uptake mechanism may explain to some extent why alveolar macrophages (via phagocytosis by forming large phagosomes) take up the majority of MVs in the alveolar space compared to epithelial cells (via clathrin and caveolin-dependent endocytosis by forming much smaller plasma membrane vesicles).

Since MVs express PS on their surface and integrins have been implicated in the uptake of apoptotic material *via* binding of PS, we investigated the expression of various integrin receptors in these alveolar cells. We found that alpha V beta III was the only integrin expressed by murine epithelial cells whilst alveolar macrophages expressed both MERTK and alpha V beta III. Inhibition of integrin/PS binding with annexin V substantially blocked epithelial cell uptake while alveolar macrophage uptake was moderately inhibited. We also investigated the expression of scavenger receptors on both cell types, and as expected, identified several on alveolar macrophages, particularly MARCO (class A) and CD36 (class B), both of which have been shown to play a prominent role in removing apoptotic material (50, 51) and particles (32, 52, 53), but not on epithelial cells. Polyinosinic acid, a known inhibitor of scavenger receptors particularly class A (33, 54), markedly reduced MV internalization in macrophages whilst having little effect on epithelial cells. These results suggest that integrin/PS binding (specifically *via* alpha V beta III) plays a crucial role in MV uptake by epithelial cells, while MV uptake by alveolar macrophages are largely dependent on scavenger receptors but integrin/PS binding may also play a role.

There are some caveats to our work. We used a membrane-bound dye DiD to measure MV uptake, but this dye may affect the functional structure of MVs. However, the use of fluorescent markers is the current gold standard method of visualizing MV internalization, and these stained MVs had similar pro-inflammatory effects to un-stained MVs used in our previous studies (9). There is also a possibility that DiD may leak from stained MVs and cells, falsely conveying uptake in recipient cells. However this is unlikely since alveolar macrophages were preferentially stained with DiD, compared to epithelial cells which would be uniformly stained if DiD was truly leaking from vesicle membranes. In addition to this, uptake was inhibited by low temperature, endocytosis and receptor-mediated uptake inhibition, making significant DiD leak or artefactual uptake of precipitated DiD by cells very unlikely. Furthermore, whilst we have presented a comprehensive evaluation of MV uptake mechanisms within the alveolar space, this was based on pharmacological inhibitor studies and more specific evidence would be required to confirm the molecular pathways. For example, scavenger receptors MARCO and CD36 have been implicated in EV uptake previously (55, 56), but it was beyond the scope of the study to precisely define whether these specific scavenger receptors were responsible for initiating MV endocytosis. Further studies, potentially using MARCO or CD36 knockout mice/SiRNA knockdown cells, would be prudent to elicit if alveolar macrophages were reliant on a particular scavenger receptor during MV internalization.

On the other hand, our data have a number of strengths as a study investigating MV interaction with target cells within the lungs. Firstly, we have investigated MV uptake in the alveolus as a whole, rather than just concentrating upon individual alveolar cells. We have comprehensively characterized MV processing in the alveolar space using both *in vitro* and *in vivo* models, while previous studies have just investigated internalization using *in vitro* models (19, 24). Furthermore, we have used MVs from different sources, both *in vitro* and *in vivo* generated, to demonstrate that alveolar macrophages take up the majority of MVs irrespective of their phenotype or origin (19, 23, 24). Finally, we have employed robust methodologies to reliably assess MV uptake by different cells in these models, e.g. reproducible standardization of administered MV doses using fluorescence, sensitive quantification of MV uptake by individual cells using flow cytometry, morphological confirmation of MV internalization by confocal microscopy, and mechanistic investigations into MV uptake using combined surface marker assessment/inhibition experiments.

In conclusion, this study demonstrates, for the first time, that alveolar macrophages internalize the majority of MVs within the alveolar space under resting conditions, but during intra-alveolar inflammation, MV uptake by epithelial cells is substantially increased, leading to MV-mediated alveolar epithelial activation. Furthermore we have established that alveolar macrophages and epithelial cells internalize MVs *via* contrasting mechanistic pathways: alveolar macrophages predominantly engulf MVs *via* scavenger receptors, whilst epithelial cells internalize MVs through a PS/integrin receptor-mediated endocytosis pathway. These data elucidate crucial mechanistic information describing how MVs can produce alveolar epithelial injury, exacerbating lung inflammation and have key implications when understanding MV signaling within the alveolar space and MV interactions with target cells. As potential novel therapeutic targets, these data highlight future areas of study by which researchers can modulate the actions of MVs within the alveolar space.

## Data Availability Statement

The original contributions presented in the study are included in the article/**Supplementary Material**. Further inquiries can be directed to the corresponding author.

## Ethics Statement

The animal study was reviewed and approved by Ethical Review Board of Imperial College London.

## Author Contributions

SS and MT contributed to the study design, protocol and study materials. SS, KO’D, EA, MK, SVS, and PS contributed to collection of data. SS and MT performed the statistical analysis. SS, MW, and MT wrote the first draft of the manuscript. All authors contributed to interpretation of the data.

## Funding

P54008: Medical research Council and British Journal of Anaesthesia; P86239: Academy of Medical Sciences; Chelsea and Westminster Health Charity.

## Conflict of Interest

The authors declare that the research was conducted in the absence of any commercial or financial relationships that could be construed as a potential conflict of interest.

## Publisher’s Note

All claims expressed in this article are solely those of the authors and do not necessarily represent those of their affiliated organizations, or those of the publisher, the editors and the reviewers. Any product that may be evaluated in this article, or claim that may be made by its manufacturer, is not guaranteed or endorsed by the publisher.
