# Supplementary material for: Microvesicle-Mediated Communication Within the Alveolar Space: Mechanisms of Uptake by Epithelial Cells and Alveolar Macrophages
Source: Front Immunol. 2022 Apr 27;13:853769. doi: 10.3389/fimmu.2022.853769 (PMC9094433; doi:10.3389/fimmu.2022.853769)
Supplement: Supplementary file 1 [file DataSheet_1.pdf]

Supplementary Figure 1.

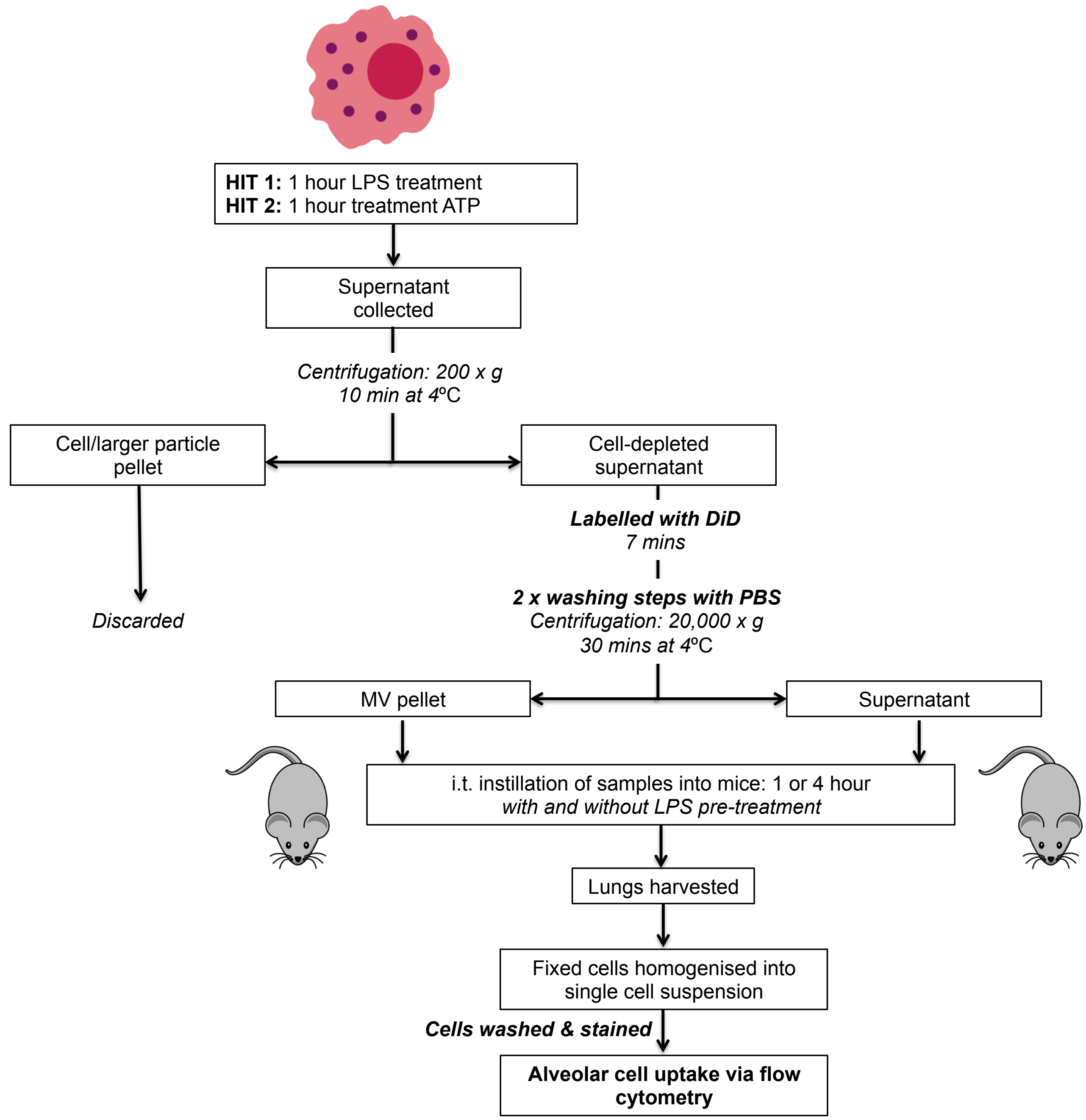

Supplementary Figure 1. A standardized amount of DiD labeled fluorescent MVs (25,000 relative florescence units (RFU)) was instilled into the trachea of mice with and without 20ng LPS pre-treatment. Either 1 or 4 hours after instillation mice were euthanized, lungs were removed and homogenized into single cell suspension. Uptake was then evaluated by assessing mean fluorescence intensity (MFI) of DiD in alveolar cells by flow cytometry.

Supplementary Figure 2.

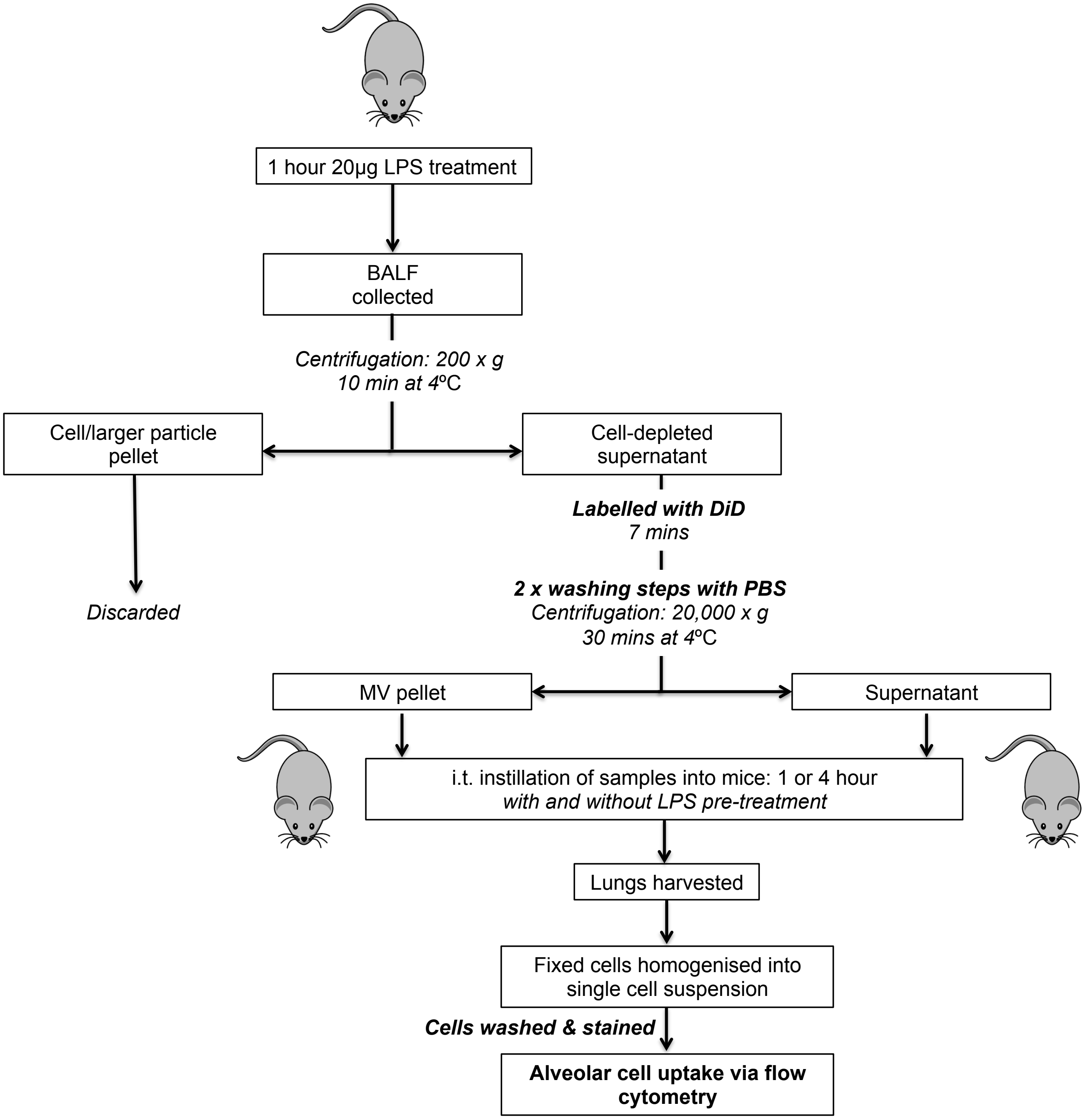

Supplementary Figure 2. We isolated intra-alveolar MVs from an in vivo model of acute lung injury (ALI), which were composed of 'mixed' populations of primary cell-derived MVs (predominantly composed of alveolar macrophage and epithelial-derived MVs) and have significant pro-inflammatory activity. A standardized amount of these DiD labeled fluorescent intra-alveolar MVs (25,000 relative florescence units (RFU)) were instilled into the trachea of mice with and without 20ng LPS pre-treatment. Either 1 or 4 hours after instillation mice were euthanized, lungs were removed and uptake was then evaluated by assessing mean fluorescence intensity (MFI) of DiD in alveolar cells by flow cytometry.

Supplementary Figure 3.

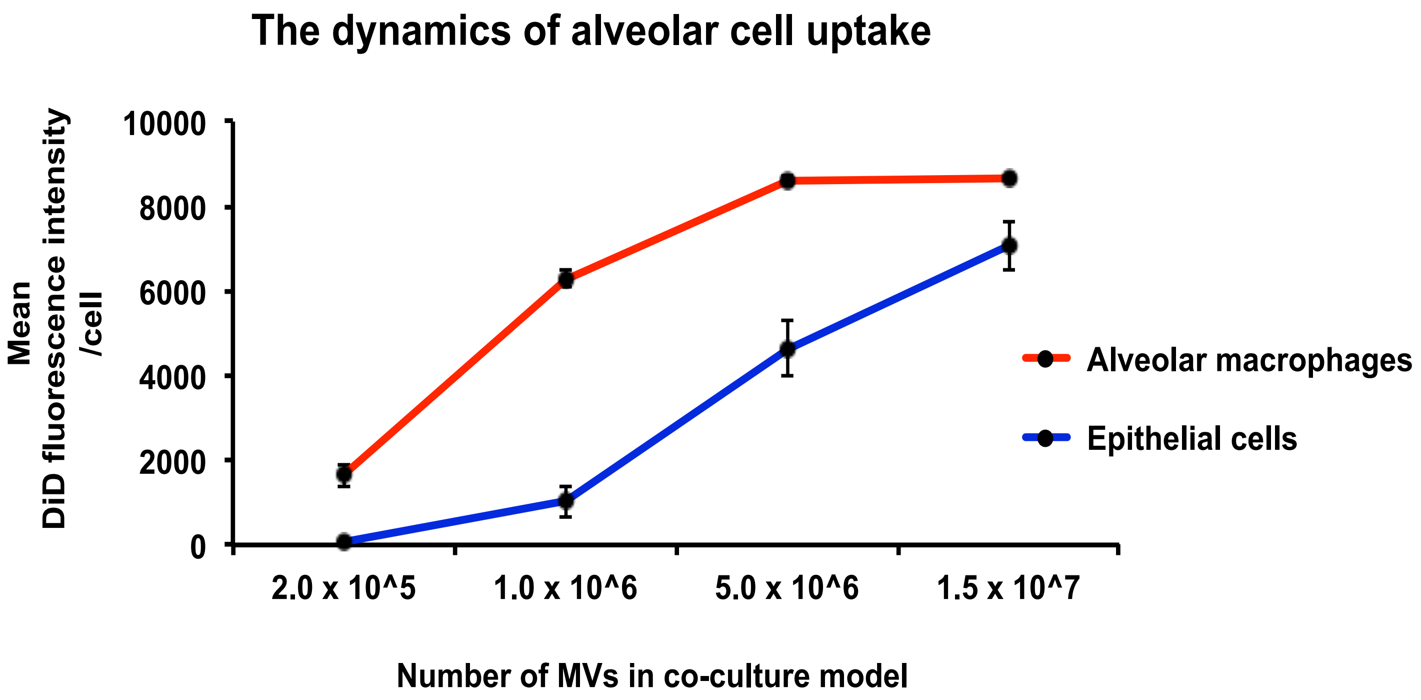

Supplementary Figure 3. The effect of increasing MVs on alveolar macrophage and epithelial cell uptake in our co-culture system after 4 hours incubation. Alveolar macrophages appeared to become saturated and unable to internalize any further MVs. At this point, epithelial cells started taking up MVs to almost a similar extent as alveolar macrophages, presumably as there is less competition from alveolar macrophages. It is important to note though that the number of MVs required to precipitate this effect far exceeds the number of macrophage MVs measured in BALF in our in vivo models of ALI.
